# Effects of exercise intervention on body morphology in obese college students: a systematic review and meta-analysis

**DOI:** 10.3389/fpubh.2025.1595862

**Published:** 2025-05-22

**Authors:** Wengao Liao, Wenlu Yu, Yuanji Zhao, Zonglin Ma, Jie Xie

**Affiliations:** ^1^School of Physical Education, Wuhan Sports University, Wuhan, Hubei, China; ^2^College of Physical Education, Chengdu University, Chengdu, Sichuan, China; ^3^College of Physical Education, Civil Aviation Flight University of China, Deyang, Sichuan, China; ^4^School of Physical Education, Tianfu College, Southwestern University of Finance and Economics, Mianyang, Sichuan, China

**Keywords:** health, exercise, obese college students, meta-analysis, systematic review

## Abstract

**Background:**

Obesity, as a public health disease, is prevalent among college students and seriously affects their quality of life and academic performance. Timely intervention through an effective exercise program is of great significance in improving the physical health status of obese college students and preventing the emergence of obesity complications. Due to the lack of a systematic review of the effects of exercise intervention on the body shape of obese college students, this study aims to systematically evaluate the effects of exercise intervention on the body shape of obese college students, to provide a reference for the development of an effective exercise intervention program for the group of obese college students.

**Methods:**

Five databases, Web of Science, PubMed, Embase, MEDLINE, and The Cochrane Library, were searched for randomized controlled trials (RCTs) on the effects of exercise interventions on obese college student populations from the date of establishment. The methodological quality of the included literature was assessed using the Rob 2.0 risk of bias tool, and then meta-analysis, subgroup analysis, and sensitivity analysis were performed using Review Manager software version 5.4.

**Results:**

A total of 12 original studies with a sample size of 961 individuals were included in this study. Meta-analysis showed that exercise intervention had a statistically significant effect on obese college students’ BMI [SMD = −0.42, 95% CI (−0.80, −0.04), *p* = 0.03], waist circumference [SMD = −0.61, 95% CI (−1.04, −0.18), *p* = 0.005] and hip circumference [SMD = −0.72, 95% CI (−1.40, −0.04), *p* = 0.04], and may be limited by factors such as intervention period, intervention duration, number of interventions, and type of interventions, Exercise interventions had a statistically significant effect on obese college students’ body fat percentage [SMD = −0.33, 95% CI (−0.79, 0.13), *p* = 0.16] and waist-to-hip ratio [SMD = −0.34, 95% CI (−0.89, 0.22), *p* = 0.23] did not have a significant intervention effect. Subgroup analyses showed that overall the intervention effect of ≥12 weeks was better than 8 weeks; the intervention effect of 30–60 min/revision was better than <30 min/revision and >60 min/revision; the intervention effect of exercise <5 times per week was better than ≥5 times per week; and the intervention effect of exercise in combination with aerobic combined with resistance training and integrative training was better than that of performing aerobic exercise alone.

**Conclusion:**

Exercise interventions have a positive effect on weight loss and improved body shape in obese college students.

**Systematic review registration:**

PROSPERO, CRD420251012259, https://www.crd.york.ac.uk/PROSPERO/view/CRD420251012259.

## 1 Introduction

Obesity and overweight are common health problems around the world (1). According to the World Health Statistics Report, as of 2022, a total of 2.5 billion adults (18 years old and above) are overweight globally, of which 890 million people suffer from obesity, and this trend is expected to continue to rise in the future (2, 3). Obesity can be divided into bad habits or congenital genetic factors that lead to simple obesity, drug use, or certain diseases triggered by secondary obesity, generalized obesity central obesity, and other types (4, 5). In daily life, observing the waist circumference, hip circumference body fat distribution, and other body shape indicators, is one of the common methods to assess health status, but it needs to be combined with other clinical indicators for a comprehensive analysis (6, 7). BMI as a commonly used indicator for the diagnosis of obesity, because of its simple, convenient characteristics in the clinic has been widely quoted, but by the influence of factors such as age, gender, and ethnicity, the use of the BMI index to determine the individual’s obesity status alone has some limitations. BF% is one of the most important indicators for determining obesity and refers to the percentage of body fat content in total body weight. Excessive hip circumference and waist-to-hip ratio are also considered unhealthy body forms caused by obesity (8). In addition, the International Diabetes Federation (International Diabetes Federation, IDF) pointed out that the waist circumference is the evaluation of the standard indicators of central obesity, waist circumference is too large for obese patients whose abdominal visceral fat content can reach 2–3 times that of a normal adult, compared with normal people are more likely to suffer from high blood pressure and early cardiovascular diseases such as heart muscle (9).

The college student group is the future builder and guardian of the future of the country, which is an important guarantee to enhance the competitiveness of the country and realize sustainable development. Under the influence of long-term sedentary, lack of exercise unhealthy dietary habits, and other bad life patterns (10), college student groups lead to the accumulation of fat in the subcutaneous tissue, the accumulation of visceral fat intensifies (11), and the efficiency of energy metabolism decreases (12, 13), thus triggering obesity. To obtain rapid weight loss, most obese college students use dieting or drug intervention to lose weight, although they can obtain obvious slimming effects in a short period, the rebound effect and side effects of drugs lead to difficulties in maintaining the weight loss effect of college students (14). Obesity not only poses a threat to the physical health of college students but also adversely affects their psychological state, leading to hypertension, diabetes, anxiety, depression, and other clinical or psychological diseases (15). Some studies have shown that the obesity rate of college students is increasing year by year, and the proportion of overweight and obesity among college students has reached more than 30% (16, 17). Therefore, the problem of obesity among college students should be given enough attention.

Exercise can not only improve the activity of lipolytic lipase in the body, accelerate the lipolysis function, and improve the utilization of fat by the body, but also significantly enhance the catecholamine level of the exercisers, thus promoting the oxidation of adipose tissue, which is one of the effective interventions to reduce body weight (18). Existing studies have confirmed the existence of the influence of exercise intervention on the weight loss effect of college student groups. For example, cycling combined with BFRT can help improve the body composition and blood lipid levels of obese college students (19), aerobic exercise of different intensities produces different changes in the body composition of obese patients (20), and aerobic exercise combined with resistance exercise is beneficial to the increase of muscle strength while reducing weight (21). Some studies have concluded that high-intensity interval training (HIIT) yields better fat loss compared to moderate-intensity continuous training (MICT) (16, 22). College students must engage in physical exercise to gain good physical fitness and reduce the chance of developing overweight or obesity (23).

Although the effect of exercise on the treatment and prevention of obesity has been confirmed, most of the previous studies only describe the effect of exercise intervention on the relevant body indicators of obese patients, and rarely discuss the efficiency and trend of the changes in the various body indicators, which is not conducive to the development of the optimal exercise program suitable for the condition of obese patients, thus affecting the effect of weight loss. In addition, there are insufficient review studies on the changes in body morphology of the special group of obese college students by exercise intervention, which is not conducive to obtaining scientific guidance on exercise intervention for the group of obese college students. Therefore, the purpose of this study is to conduct a systematic evaluation through meta-analysis, to explore the effects of physical exercise on improving the body morphology of obese college students, to analyze the characteristics of the changes in various body morphology indexes, and based on the results of the study, to propose better exercise recommendations for helping obese patients in controlling their body morphology.

## 2 Methods

This study followed the Preferred Reporting Items for Systematic Evaluation and Meta-Analysis (PRISMA) guidelines (24). Registered through the International Registry for Prospective Systematic Evaluation (PROSPERO). (registration number CRD420251012259).

### 2.1 Search strategy

Relevant literature up to February 20, 2025, was searched in five English-language databases: Web of Science, PubMed, Embase, MEDLINE, and The Cochrane Library. The search strategy followed the PICOS principle of medical searching: (P) Study population: obese college students; (I) Intervention: exercise and exercise; (C) Comparative measures: exercise intervention group and control group without the intervention; (O) Outcome indicators: BMI, percent body fat, waist circumference, hip circumference, and hip-to-waist ratio; and (S) Type of study: RCTs.

### 2.2 Selection criteria

To ensure the scientific validity and effectiveness of the included literature, 2 authors (WL and WY) reviewed and screened the results of the literature search, and in case of disagreement, a third author (YZ) was appropriately consulted to reach a consensus on whether to include the literature. Literature inclusion criteria should include (1) the type of studies included in the literature must be randomized controlled clinical trials (RCTs); (2) the study subjects must meet the WHO criteria for obesity and overweight (BMI ≥ 25 kg/m2) for college students (Male and Female), and the study subjects must be free of other illnesses, and (3) an exercise intervention was applied to the experimental group, and the control group compared to the experimental group was applied the normal exercise intervention or no intervention was imposed and lived a normal life. (4) The included studies included at least one indicator of body shape of college students: body mass index (BMI), body fat percentage (BF%), waist circumference (WC), hip circumference (HC), and waist-to-hip ratio (WHR).

Exclusion criteria included (1) literature that did not satisfy any 1 of the inclusion criteria, (2) study types that were not randomized controlled trials, (3) duplicates, reviews, and conference papers, and (4) literature with poorly described and incomplete experimental data.

### 2.3 Data extraction

To ensure the accuracy and consistency of data extraction, literature that met the inclusion criteria was read in full by 2 authors (WL and WY) and data were extracted using a standardized form. In case of disagreement, the 3rd author (YZ) was consulted and uncertainties were resolved through discussion and negotiation. The extracted information included: (1) basic information: first author, year of publication, nationality; (2) experimental characteristics: experimental sample size, sample gender, sample age, intervention period, intervention measures; (3) outcome indicators.

### 2.4 Risk of bias

The quality of the literature was assessed using the Rob 2.0 risk of bias assessment tool, which evaluates the methodological quality of the included literature in five modules: bias arising from the randomization process, bias from deviation from established interventions, bias from missing outcome data, bias from outcome measurement, and bias from selective reporting of outcomes. Alternative answers to each signaling question were (Yes, Y), (Probably yes, PY), (Probably no, PN), (No, N), and (No information, NI), and individuals were rated using low risk of bias, probable risk, and high risk of bias ratings. Based on the final evaluation results, a risk of bias map and a risk of bias summary map were generated in the Rob 2.0 risk of bias assessment tool.

### 2.5 Statistical analyses

All analyses were performed using Review Manager software version 5.4 strictly following PRISMA guidelines. Since the outcome indicators included in this study were continuous variables and the units of test for the included results were inconsistent, the standardized mean difference (SMD) and confidence interval (95% CI) were used as the effect scales to calculate the combined effect sizes. *p*-values were based on the test of significance with a range of 0 to 1. When *p* < 0.01, it indicated that the analyzed results were highly significant, and when 0.01 ≤ *p* < 0.05, the results of the meta-analysis could be considered that the analyzed results are significantly different and the meta-analysis results are statistically significant. On the contrary, when *p* > 0.05, there is no statistical significance. According to the Cochrane Handbook of Systematic Evaluation reporting standards, I^2^ represents the level of heterogeneity between studies, and the value range is 0–100%, when I^2^ = 0%, there is no heterogeneity between studies; when 0 < I^2^ ≤ 50%, there is a negligible lower heterogeneity between studies, and a fixed-effects model is selected for meta-analysis; when I^2^ > 50%, it indicates that there is a non-negligible heterogeneity, which should be analyzed using a random-effects model, and the source of heterogeneity should be determined by meta-regression analysis, subgroup analysis, and sensitivity analysis.

## 3 Results

### 3.1 Literature search results

The study was cross-searched in the database using the strategy of subject words + free words, and a total of 3,751 documents were retrieved. The literature data obtained from the search was imported into the Endnote X9 literature management tool, and 1,463 duplicates were excluded; the titles and abstracts of the remaining literature were browsed by 2 authors, and the titles and abstracts of the remaining literature were excluded to exclude the non-randomized controlled experiments, the non-obese populations, the non-college student populations, and the study types that did not meet the inclusion criteria, The total number of literature that did not meet the inclusion criteria, such as incompatible study objectives and incompatible outcome indicators, was 2,242; then we read the full text of the literature and excluded 12 papers with missing experimental data; in addition, we judged the papers with irrational experimental design and those without full text content as low-quality papers, and excluded a total of 14 papers with irrational experimental design and 8 papers without full text content, and finally 12 papers were included in the meta-analysis, and the process of literature screening is shown in Supplementary Figure 1.

### 3.2 Study characteristic

The basic characteristics of the 12 papers included in the study are shown in Supplementary Table 1. Most of these studies were from Asia (25, 26, 36–43, 45, 46), with only one study from North America (44). The study subjects were all college students who met the WHO criteria for overweight and obesity, totaling 961, including 537 in the experimental group and 424 in the control group; most of the studies originated from Asia, with an intervention period of 8–16 weeks, and one case was not reported; the maximum sample size of a single session of the experiments included in the literature was 300, with a minimum of 16; and the exercise interventions were based on AE and RT training. All reported the outcome indicators required for the study.

### 3.3 Risk of bias evaluation

Assessment of the 12 papers included in the meta-analysis using the Rob 2.0 risk of bias assessment tool showed that 5 papers were assessed as possibly at risk or high risk of bias in randomization and 2 papers were assessed as possibly at risk of bias in deviation from the established interventions; none of the 12 papers were at risk of bias in missing endpoint data and endpoint measurements; and 2 papers were assessed as possibly at risk of bias in selective reporting of outcomes. Bias was rated as possibly at risk. Supplementary Figure 2 shows that the included literature is at some risk of bias.

### 3.4 Meta-analysis results

#### 3.4.1 Meta-analysis of body mass index (BMI)

Supplementary Figure 3 shows that out of the 12 literature included, a total of 11 studies were conducted on the effect of exercise interventions on the BMI of obese college students, including 437 in the experimental group and 324 in the control group. The results of the heterogeneity test (*I*^2^ = 80%) indicated a high degree of heterogeneity among the studies, so meta-analysis was performed using the random effects model, and the results showed that the combined effect size [SMD = −0.42, 95% CI (−0.80, −0.04), *p* = 0.03] was statistically significant. There was a significant difference between the experimental group and the control group, and the BMI index of obese college students could be significantly reduced by exercise intervention.

#### 3.4.2 Meta-analysis of percent body fat (BF%)

Supplementary Figure 4 shows that a total of 11 of the 12 papers included in the analysis examined the effect of exercise interventions on BF% in obese college students, including 496 in the experimental group and 387 in the control group. The results of the heterogeneity test (*I*^2^ = 88%), which showed a high degree of heterogeneity between studies, and meta-analysis using a random effects model indicated that the combined effect size [SMD = −0.33, 95% CI (−0.79, 0.13), *p* = 0.16] was not statistically significant. There was no significant difference between the experimental and control groups, and the exercise intervention did not achieve a significant effect on reducing BF% in obese college students.

#### 3.4.3 Meta-analysis of waist circumference (WC)

Supplementary Figure 5 shows that a total of 8 of the 12 papers included in the analysis examined the effects of exercise interventions for WC in obese college students, including 372 in the experimental group and 289 in the control group. The results of the heterogeneity test were (*I*^2^ = 80%), there was a high degree of heterogeneity among the studies, and meta-analysis should be carried out using the random effects model, and the results showed that the combined effect size [SMD = −0.61, 95% CI (−1.04, −0.18), *p* = 0.005] was statistically significant. Exercise intervention had a significant effect on waist circumference reduction in obese college students.

#### 3.4.4 Meta-analysis of hip circumference (HC)

Supplementary Figure 6 shows that among the 12 papers included in the analysis, a total of 5 papers studied the effects of exercise interventions for HC in obese college students, including 327 in the experimental group and 259 in the control group. The results of the heterogeneity test were (*I*^2^ = 92%), there was a high degree of heterogeneity between studies, and a random effects model should be used for meta-analysis, and the results showed that the combined effect size [SMD = −0.72, 95% CI (−1.40, −0.04), *p* = 0.04] was statistically significant. Exercise intervention significantly reduces hip circumference in obese college students.

#### 3.4.5 Meta-analysis of waist-to-hip ratio (WHR)

Supplementary Figure 7 shows that a total of 7 of the 12 papers included in the analysis examined the effects of exercise interventions in obese college students with WHR, including 441 in the experimental group and 368 in the control group. The results of the heterogeneity test were (*I*^2^ = 92%), there was a high degree of heterogeneity among the studies, and meta-analysis should be carried out using the random effects model, which showed that the combined effect size of [SMD = −0.34, 95% CI (−0.89, 0.22), *p* = 0.23] was not statistically significant and that the exercise intervention on the improvement of WHR in obese college students did not be able to achieve a significant effect.

### 3.5 Subgroup analyses

The intervention effect of an exercise intervention on the body morphology of obese college students was influenced by a variety of factors, and to further explore the sources of heterogeneity in the study, subgroup analyses were conducted to analyze the factors that might cause heterogeneity. The effect of exercise intervention on body morphology in the obese college student population is closely related to the type of exercise intervention based on intervention cycle, single intervention duration, weekly intervention frequency, and exercise intervention type, according to the characteristics of the literature included in the meta-analysis, the intervention cycle was divided into 2 subgroups of 8 and ≥12 weeks, the duration of single intervention was divided into 3 subgroups of ≤30 min/times, 30-60 min/times, >60 min/times, and the type of exercise intervention was divided into 3 subgroups of <5 times and ≥5 times. 60 min/session 3 subgroups, weekly intervention frequency was categorized into <5 and ≥5 subgroups, and the type of exercise intervention was categorized into 3 subgroups: AE, AE + RT, and other interventions. In addition, literature without specific descriptions of intervention time and intervention frequency was excluded to ensure the validity of the results of the subgroup analysis.

#### 3.5.1 Subgroup analysis of intervention cycles

Supplementary Table 2 shows the results of the subgroup analysis of different intervention cycles on various indicators of body morphology in obese college students. Except for the 8-week exercise intervention subgroup where the heterogeneity of the effect of the intervention on BMI (*I*^2^ = 48%) and WHR (*I*^2^ = 50%) became non-significant, the heterogeneity of the remaining subgroups remained significant, indicating that the intervention cycle was not an influencing factor leading to higher heterogeneity among the studies.

#### 3.5.2 Subgroup analysis of intervention time

Supplementary Table 3 shows the results of subgroup analyses of different intervention durations on various indicators of body morphology in obese college students. There was no heterogeneity in the effect of >60 min/session of an exercise intervention on BMI and BF% of obese college students and 30–60 min/session of an exercise intervention on HC (*I*^2^ = 0%). In contrast, the heterogeneity of the effect of the other subgroups on the effect of each indicator remained significant, indicating that the duration of the intervention was not responsible for the high heterogeneity that emerged.

#### 3.5.3 Subgroup analysis of intervention frequencies

Supplementary Table 4 shows the results of subgroup analyses of different intervention frequencies on various indicators of body morphology in obese college students. The heterogeneity of the effect of exercise interventions ≥5 times per week on the effect of WC (*I*^2^ = 15%) and the heterogeneity of the effect of exercise interventions <5 times per week on the effect of HC (*I*^2^ = 0%) became non-significant. In contrast, the heterogeneity of the effect of the other subgroups of intervention frequency on the effect of each indicator remained significant, suggesting that the frequency of intervention was not the source of their heterogeneity.

#### 3.5.4 Subgroup analysis of intervention types

Supplementary Table 5 shows the results of subgroup analyses of different intervention types on various indicators of body morphology in obese college students; the heterogeneity of the effect effects of using other exercise interventions on BMI, the effect of AE + RT interventions and other exercise interventions on WC, and the effect of AE interventions on HC became non-significant (*I*^2^ = 0%), and the heterogeneity of the effect of AE interventions on BF% (*I*^2^ = 29%) and the effect of AE heterogeneity of the effect of the +RT intervention on the effect of WHR (*I*^2^ = 41%) similarly became nonsignificant. However, no heterogeneity became nonsignificant for all 3 subgroups under any of the metrics, suggesting that again the type of exercise intervention was not responsible for the high heterogeneity seen across studies.

### 3.6 Sensitivity analysis

To investigate whether the heterogeneity among studies was caused by a single literature, sensitivity analysis was performed using Review Manager software. Supplementary Table 6 shows the results of the sensitivity analyses. After the article-by-article elimination of the data from the literature included in the meta-analysis of BMI, BF%, WC, HC, and WHR, respectively, the SMD combined effect sizes of all the studies were within the range of 2 confidence intervals, and no statistical changes were affecting the results of the analyses, and the results of the studies had a certain degree of stability and reliability. However, the heterogeneity of Chih-Hui Chiu’s study (2017) in the sensitivity analysis of the literature related to BMI, WC, and WHR and Wenjiang Liu’s study (2023) in the sensitivity analysis of the literature related to HC appeared to be significantly lower, suggesting that this literature may be the cause of the higher heterogeneity of the findings and that it is necessary to examine the content of the literature once again to determine whether or not to include them into the meta-analysis.

## 4 Discussion

This systematic review explores the effects of exercise interventions on body shape-related indicators of obese college students by summarizing data from 12 randomized controlled trials. The results of the study show that exercise intervention can effectively reduce the obesity symptoms of college students and have an impact on their related body shape indexes, in which the effect of exercise intervention on BMI, waist circumference, and hip circumference of the group of obese college students is better than that of percentage of body fat and waist-to-height ratio. Exercise intervention did not have a significant effect on the reduction of body fat percentage and waist-height ratio of obese college students, and there was a high degree of heterogeneity among the studies. The reasons for the high heterogeneity between the studies and the overall heterogeneity of the articles need to be discussed.

By comparing and analyzing the experimental procedures and pre-and post-trial data on the percent body fat and waist-to-height ratio included in the meta-analysis literature, it was found that the results of the failure of exercise interventions to significantly reduce percent body fat and waist-to-height ratio in obese college students may be affected by a variety of factors, such as insufficient duration and intensity of exercise interventions, different types of exercise interventions, and experimental sample sizes. First, the duration of exercise intervention in randomized controlled trials using body fat percentage and waist-to-height ratio as outcome indicators is mainly 30–60 min each time, while some studies have shown that a single longer exercise intervention is required to affect the reduction of visceral fat accumulation and the reduction of body fat percentage and waist-to-height ratio in obese patients (27). Secondly, low intervention frequency leading to insufficient exercise intensity is one of the most important reasons affecting the reduction of body fat percentage and waist-to-height ratio, with most of the literature having less than five exercise interventions, which fails to create a calorie gap and thus affects weight loss. Finally, in the literature involving body fat percentage and waist-to-height ratio, most of them used AE as the main exercise intervention and lacked resistance training and high-intensity interval training interventions, which led to a slow increase in muscle mass and difficulty in increasing basal metabolic rate in obese college students, thus affecting the weight loss effect.

Regarding the meta-analysis results presenting high heterogeneity, this paper identifies the literature that may cause high heterogeneity through sensitivity analysis and carefully reads and analyzes the content of the literature to explore the source of heterogeneity. The sensitivity results showed that two papers by Chih-Hui Chiu and Wenjiang Liu may be the source of high heterogeneity in the study results. Further analysis of the articles revealed that Chih-Hui Chiu’s study was conducted by <12 weeks, <5 times per week, 30–60 min of AE intervention on obese college students’ BMI, BF%, WC, HC, and WHR The effect of the intervention was measured and yielded findings that were consistent with other literature and met the inclusion criteria, so the literature was still included in the meta-analysis, and the single type of intervention and less frequent interventions may be the reason for the higher heterogeneity caused by this literature. Wenjiang Liu’s study, which examined the effects of AE and RT interventions on BMI, BF%, WC, HC, and WHR through AE and RT interventions, also met the inclusion criteria, and the literature was still included in the meta-analysis; however, the failure of the literature to report on the intervention period, duration of a single intervention, frequency of weekly interventions, and subject-related characteristics increased the likelihood of causing higher heterogeneity in the study results Likelihood.

Results regarding the effect of exercise intervention on body morphology in obese college students. Regarding the intervention period, the results of meta-analysis and subgroup analysis (Supplementary Table 2) showed that the effect of exercise intervention over 12 weeks on the indicators of body morphology of obese college students was more significant, except for BF%. The lag of weight loss effect is a complex physiological and psychological phenomenon, caused by a variety of factors such as physical adaptation, diet control, exercise, metabolic adaptation, and hormonal changes in the level, which is specifically manifested in the time difference of the changes in the indicators of body shape. The change in body shape is a visual reflection of the weight loss effect (28), and due to the existence of weight loss lag, it is difficult to produce significant changes in body shape in a short period, which is consistent with the conclusion of this study that the effect of ≥12 weeks of exercise intervention is greater than that of 8 weeks of exercise intervention. Therefore, in the process of weight loss through exercise intervention, obese college students should reasonably arrange the exercise load according to their conditions, and obtain the best weight loss effect through a longer cycle of exercise intervention.

In terms of intervention duration, according to the results of subgroup analysis of single intervention duration, it was found that a single 30–60 min exercise intervention could achieve a more significant effect on BMI, WC, HC, and WHR of obese college students compared to a single <30 min and a single >60 min exercise intervention. For BF%, the effect of a single >60 min exercise intervention was better than the other two subgroups, which may be related to the frequency of interventions and the lack of breadth of exercise interventions in the literature included in this meta-analysis. It has been shown that prolonged exercise of more than 60 min is more effective in reducing visceral fat and body fat percentage (27), consistent with the results of the meta-analysis herein. Supplementary Table 3 shows that, overall, the effects of exercise interventions of different durations on the body shape-related indicators of obese college students were in the order of 30–60 min, >60 min, and <30 min. According to the principle of exercise physiology, the energy supply of the body in the early stage of exercise is dominated by glycogen (29), and when the exercise reaches about 30 min, the body has a large amount of myo-glycogen depletion or close to depletion and the oxygen supply is sufficient, the fat metabolism energy supply and output power is maximum (16) so that the exerciser can obtain effective fat loss effect. As time goes by, protein function gradually increases, and exercisers may experience the saving phenomenon of myo-glycogen and protein, which is not conducive to the oxidative consumption of fat, thus reducing the effect of exercise weight loss (30), which is consistent with the conclusion of this study that 30–60 min of exercise intervention can enable obese college students to obtain the best weight loss effect.

In terms of frequency of weekly interventions, the results of the study showed that exercise interventions performed <5 times per week were more effective in influencing the body shape of obese college students, in addition to the percentage of body fat. Some studies have shown that 3–5 times of exercise intervention per week can reach the effective threshold of fat loss (31). College students do not have enough time for physical activity under the pressure of study, so they can increase the level of post-exercise oxygen consumption through low-frequency (2–3 times per week) high-intensity interval training interventions to achieve better fat loss (32). In addition, high-frequency exercise more than 6 times per week may lead to overtraining risks such as elevated cortisol and muscle catabolism, which may inhibit the weight loss effect, but high-frequency and high-intensity exercise workouts can achieve better results in lowering the percentage of body fat (26, 33).

In terms of the type of exercise intervention, a total of 10 of the 12 papers included involved aerobic exercise intervention. The intervention effect of aerobic exercise on weight loss is widely recognized, but the process of prolonged, low-and moderate-intensity aerobic exercise is relatively monotonous, and most college students are unable to adhere to it for a long period, which makes it difficult to achieve sustained and effective weight loss. Comprehensive training, high-intensity interval training moderate-intensity continuous training, and other interventions compared with aerobic exercise in the intensity and duration of exercise on body fat there are different impact mechanisms (34), not only can increase the intensity of training, but also based on a reasonable interval time, to minimize the exercise time and achieve optimal weight loss (35), more easily accepted by the majority of student groups. The research data in this paper show that, in addition to body fat percentage and hip circumference, comprehensive training and other interventions are significantly better than aerobic training for other body shape indicators of obese college students.

Based on the results of the meta-analysis and subgroup analysis, it was concluded that the college student population could obtain better overall weight loss results through ≥12 weeks, <5 times per week lasting 30–60 min of combined training, high-intensity interval training, and moderate-intensity continuous training to improve obese body shape. In contrast, obese college students with higher BF% could obtain the best weight loss results through aerobic exercise interventions of <12 weeks, >5 times per week lasting more than 60 min.

### 4.1 Strengths and limitations

Strengths of this meta-analysis: a search of the literature database revealed that this is the first study of the intervention effect of exercise intervention on the body morphology of a special group of obese college students. The results of our study help provide a reference for obese college students to reduce their obesity status, improve their body morphology as well as formulate a reasonable exercise and weight loss program, and maximize the contribution to guaranteeing the physical and mental health development of college students.

Study limitations: (1) There are fewer current randomized controlled trial studies on the effects of exercise interventions on weight loss in obese college students, resulting in a smaller total sample size for the included studies. (2) Because most of the exercise interventions in the included literature did not determine whether or not to blind the subjects and researchers, resulting in some risk of bias in the included literature. (3) Only published studies were included in this study, and literature in the process of publication was not included, which may have had some impact on the results of the meta-analysis.

## 5 Conclusion

Exercise intervention can effectively help obese college students reduce their body weight, and the intervention effect on BMI index, waist circumference, and hip circumference of obese college students is significant, while the body fat percentage and waist-to-hip ratio are affected by a variety of factors, and the intervention effect is lower than that of other indicators. There is a high degree of heterogeneity among the studies, and after subgroup analysis and heterogeneity test, the results of this paper are considered stable and reliable. Exercise intervention is conducive to improving the body shape of obese college students, and is an effective means to protect and enhance the physical and mental health of obese college students. However, most college students stop doing physical exercise after achieving a certain weight loss effect, which leads to obesity again. Therefore, it is more important to maintain the improvement effect of exercise intervention on the obese body shape of college students. On the one hand, college students should develop a good habit of exercising every day, and maintain the weight loss effect as much as possible through continuous and stable exercise. On the other hand, the chances of re-obesity can be reduced and the physical health status can be further improved by establishing a social support system and utilizing school, family, and peer supervision.

## Data Availability

The original contributions presented in the study are included in the article/Supplementary material, further inquiries can be directed to the corresponding author.
