# Supplementary material for: Effects of exercise intervention on body morphology in obese college students: a systematic review and meta-analysis
Source: Front Public Health. 2025 May 22;13:1595862. doi: 10.3389/fpubh.2025.1595862 (PMC12138376; doi:10.3389/fpubh.2025.1595862)
Supplement: Supplementary file 1 [file Data_Sheet_1.docx]

Supplementary Material

# Supplementary Data

Supplementary Material should be uploaded separately on submission. Please include any supplementary data, figures and/or tables.

Supplementary material is not typeset so please ensure that all information is clearly presented, the appropriate caption is included in the file and not in the manuscript, and that the style conforms to the rest of the article.

# Supplementary Figures and Tables

For more information on Supplementary Material and for details on the different file types accepted, please see [here](https://www.frontiersin.org/guidelines/author-guidelines#supplementary-material).

## Supplementary Figures


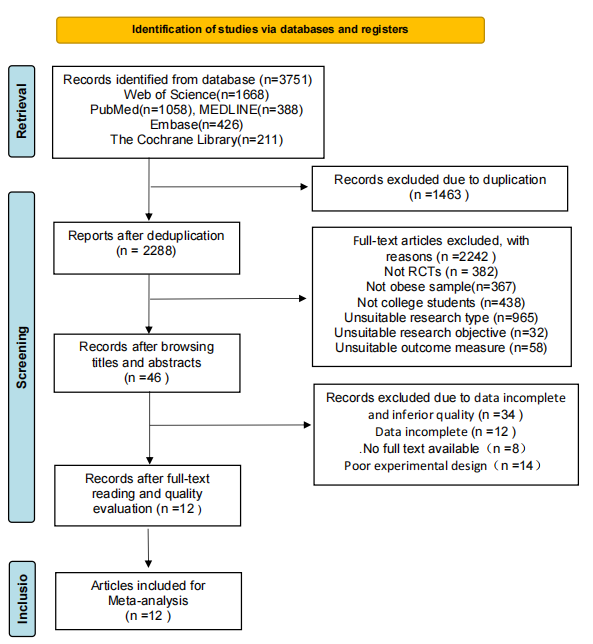


**Supplementary Figure 1.** Flow diagram of the search and study selection process.


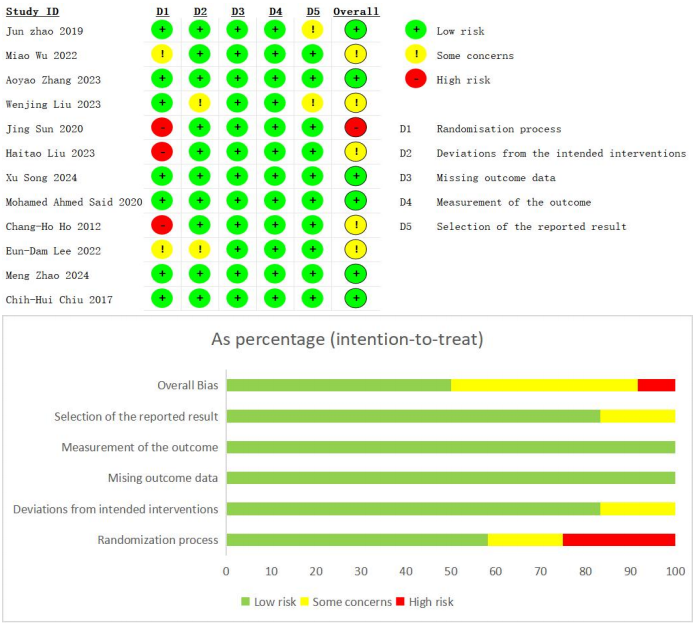


**Supplementary Figure 2.** Risk of bias graph and risk summary graph for included literature.


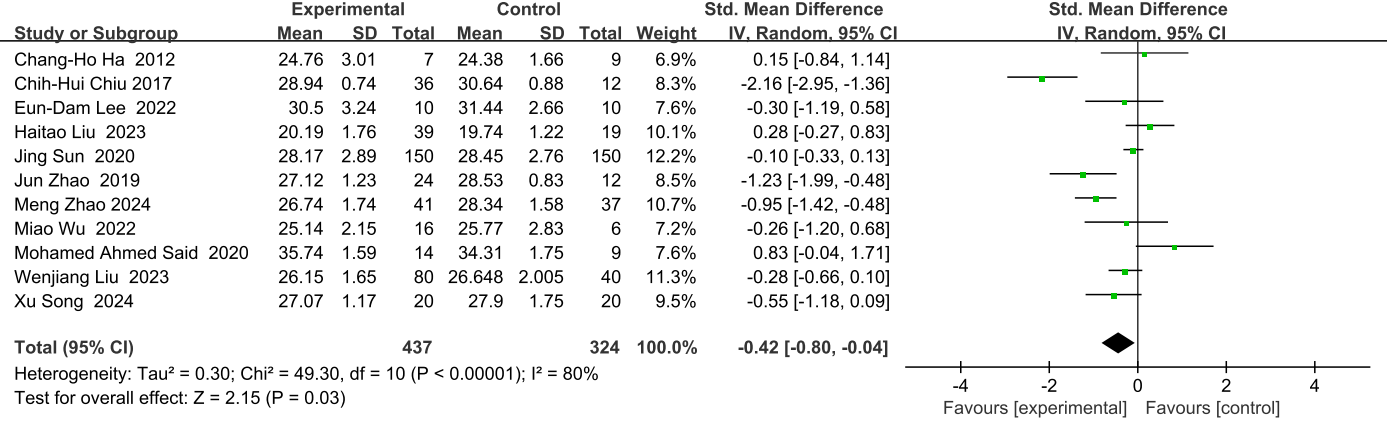


**Supplementary Figure 3.** Forest plot of the effect of exercise intervention on BMI in obese college students.


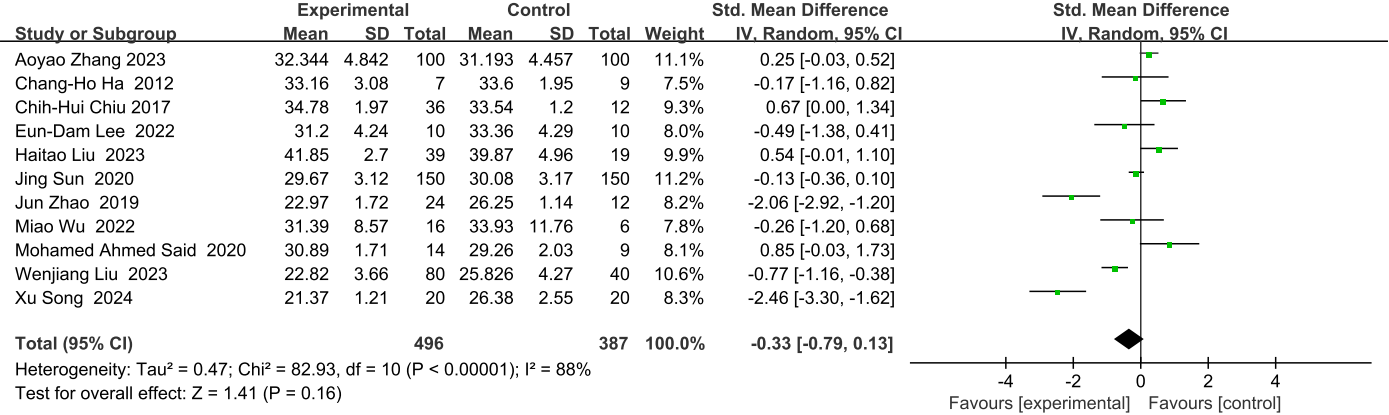


**Supplementary Figure 4.** Forest plot of the effect of an exercise intervention on BF% in obese college students.


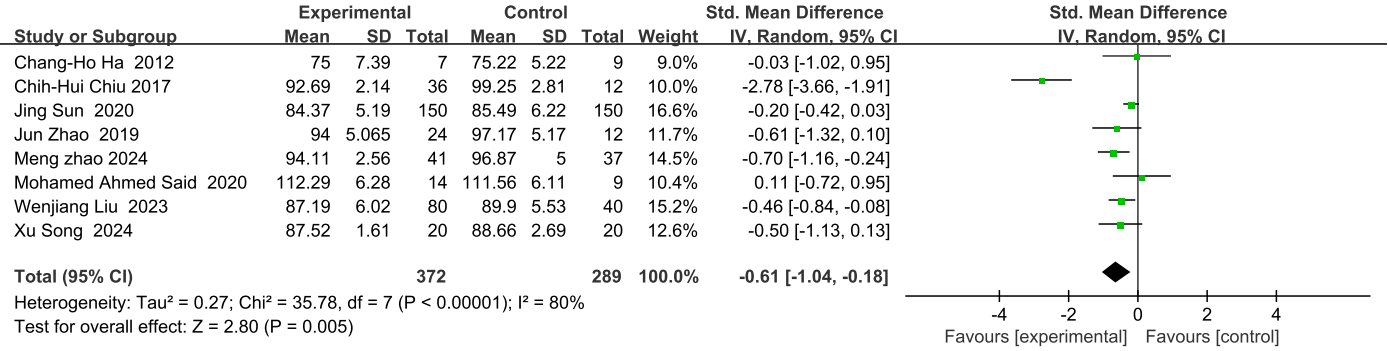


**Supplementary Figure 5.** Forest plot of the effect of an exercise intervention on WC in obese college students.


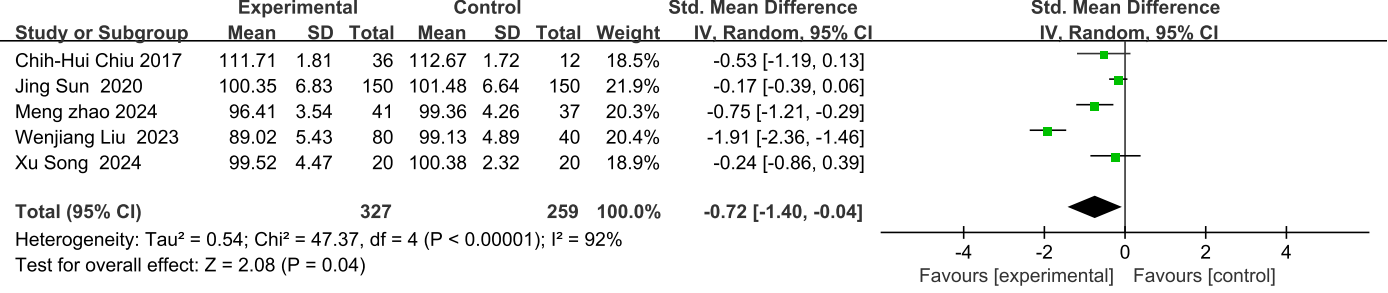


**Supplementary Figure 6.** Forest plot of the effect of an exercise intervention on HC in obese college students.


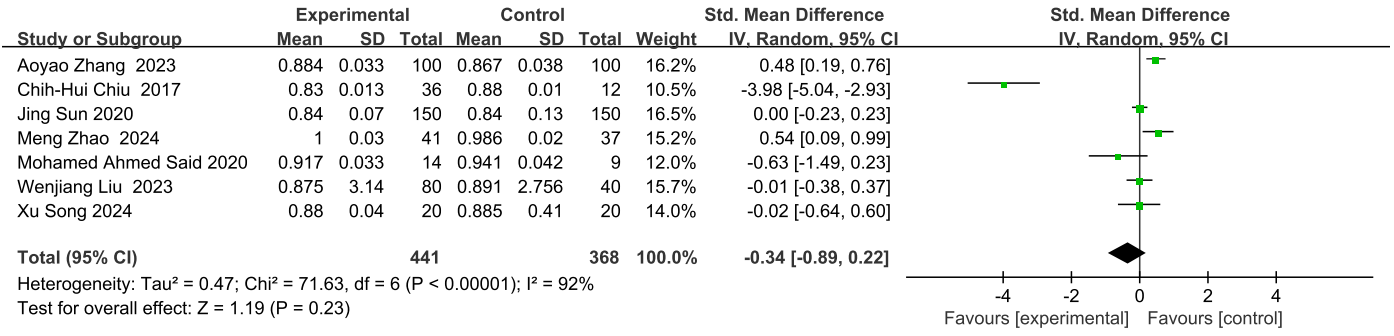


**Supplementary Figure 7.** Forest plot of the effect of an exercise intervention on WHR in obese college students.

## Supplementary Tables

| Study | Country | Duration  (weeks) | Sample  (T/C) | Gender  (M/F) | Age | Interventions | Outcomes |
| --- | --- | --- | --- | --- | --- | --- | --- |
| Jun Zhao et al.  (2019) | China | 12 | 24/12 | 36/0 | 21.52±1.04 | AE、AE+RT | ①②③ |
| Miao Wu et al.  (2022) | China | 8 | 16/6 | 8/14 | 19.00±1.00 | AE | ①② |
| Aoyao Zhang et al.  (2023) | China | 8 | 100/100 | 0/200 | 20.50±1.50 | AE、CrossFit | ②④ |
| Wenjiang Liu et al.  (2023) | China | —— | 80/40 | —— | —— | AE、RT | ①②③④⑤ |
| Jing Sun et al.  (2020) | China | 12 | 150/150 | 300/0 | 21.70±1.43 | HIIT、AE | ①②③④⑤ |
| Haitao Liu et al.  (2023) | China | 8 | 39/19 | 0/58 | 20.03±1.24 | AE | ①② |
| Xu Song et al.  (2024) | China | 8 | 20/20 | 20/20 | 21.50±3.50 | HIIT、MICT | ①②③④⑤ |
| Mohamed Ahmed  Said et al.(2020) | Saudi  Arabia | 16 | 14/9 | 23/0 | 21.50±2.50 | AE+RT | ①②③④ |
| Chang-Ho Ha et al.  (2012) | USA | 12 | 7/9 | 0/16 | 20.96±1.78 | AE、RT | ①②③ |
| Eun-Dam Lee et al.  (2022) | Korea | 12 | 10/10 | 20/0 | 25.60±1.82 | RT | ①② |
| Meng Zhao et al.  (2024) | China | 12 | 41/37 | 45/33 | 18-24 | AE | ①③④⑤ |
| Chih-Hui Chiu et al.  (2017) | China | 12 | 36/12 | 34/14 | 21.04±0.60 | AE | ①②③④⑤ |

**Supplementary Table 1.** Basic characteristics of the included literature.

| Outcomes | Amount | Subgroup  (Weeks) | SMD | 95%CI | P | Heterogeneity | | Z |
| --- | --- | --- | --- | --- | --- | --- | --- | --- |
|  |  |  |  |  |  | I^2^(%) | P |  |
| BMI | 3 | 8 | -0.14 | （-0.69，0.40） | 0.061 | 48 | 0.15 | 0.51 |
|  | 7 | ≥12 | -0.55 | （-1.17，0.07） | 0.08 | 86 | 0.01 | 1.75 |
| BF% | 4 | 8 | -0.44 | （-1.51，0.63） | 0.42 | 92 | 0.01 | 0.81 |
|  | 6 | ≥12 | -0.20 | （-0.87，0.47） | 0.56 | 84 | 0.01 | 0.59 |
| WC | 1 | 8 | -0.59 | （-1.32，0.13） | 0.12 | —— | —— | 1.57 |
|  | 6 | ≥12 | -0.68 | （-1.30，0.05） | 0.04 | 86 | 0.01 | 2.11 |
| HC | 1 | 8 | -0.24 | （-0.86，0.39） | 0.46 | —— | —— | 0.75 |
|  | 3 | ≥12 | -0.43 | （-0.84，-0.03） | 0.04 | 63 | 0.07 | 2.08 |
| WHR | 2 | 8 | 0.31 | （-0.15，0.77） | 0.18 | 50 | 0.16 | 1.33 |
|  | 4 | ≥12 | -0.91 | （-2.10，0.28） | 0.14 | 95 | 0.01 | 1.49 |

**Supplementary Table 2.** Subgroup analysis of intervention cycles.

| Outcomes | Amount | Subgroup  (Minutes) | SMD | 95%CI | P | Heterogeneity | | Z |
| --- | --- | --- | --- | --- | --- | --- | --- | --- |
|  |  |  |  |  |  | I^2^(%) | P |  |
| BMI | 1 | ≤30 | -0.10 | （-0.33，0.13） | 0.39 | —— | —— | 0.86 |
|  | 7 | 30-60 | -0.59 | （-1.25，0.07） | 0.08 | 85 | 0.01 | 1.75 |
|  | 2 | ＞60 | -0.06 | （-0.75，0.62） | 0.86 | 0 | 0.55 | 0.18 |
| BF% | 1 | ≤30 | -0.13 | （-0.36，0.10） | 0.26 | —— | —— | 0.12 |
|  | 6 | 30-60 | -0.47 | （-1.60，0.65） | 0.41 | 92 | 0.01 | 0.83 |
|  | 2 | ＞60 | -0.23 | （-0.91，0.46） | 0.52 | 0 | 0.87 | 0.65 |
| WC | 1 | ≤30 | -0.20 | （-0.42，0.03） | 0.09 | —— | —— | 1.68 |
|  | 5 | 30-60 | -0.87 | （-1.63，-0.11） | 0.03 | 84 | 0.01 | 2.23 |
|  | 1 | ＞60 | -0.03 | （-1.02，0.95） | 0.95 | —— | —— | 0.07 |
| HC | 1 | ≤30 | -0.17 | （-0.39，0.06） | 0.15 | —— | —— | 1.45 |
|  | 3 | 30-60 | -0.56 | （-0.88，-0.24） | 0.0007 | 0 | 0.43 | 3.39 |
|  | 0 | ＞60 | —— | —— | —— | —— | —— | —— |
| WHR | 1 | ≤30 | 0.00 | （-0.23，0.23） | 1 | —— | —— | 0 |
|  | 4 | 30-60 | -0.96 | （-2.52，0.60） | 0.23 | 95 | 0.01 | 1.21 |
|  | 0 | ＞60 | —— | —— | —— | —— | —— | —— |

**Supplementary Table 3.** Subgroup analysis of intervention time.

| Outcomes | Amount | Subgroup  (Times) | SMD | 95%CI | P | Heterogeneity | | Z |
| --- | --- | --- | --- | --- | --- | --- | --- | --- |
|  |  |  |  |  |  | I^2^(%) | P |  |
| BMI | 7 | ＜5 | -0.50 | （-1.14，0.15） | 0.13 | 80 | 0.01 | 1.51 |
|  | 3 | ≥5 | -0.28 | （-0.93，0.37） | 0.39 | 81 | 0.006 | 0.86 |
| BF% | 6 | ＜5 | -0.31 | （-1.30，0.69） | 0.55 | 88 | 0.01 | 0.60 |
|  | 3 | ≥5 | -0.48 | （-1.53，0.57） | 0.37 | 92 | 0.01 | 0.89 |
| WC | 5 | ＜5 | -0.78 | （-1.62，0.06） | 0.07 | 85 | 0.28 | 1.73 |
|  | 2 | ≥5 | -0.26 | （-0.55，0.03） | 0.08 | 15 | 0.01 | 1.64 |
| HC | 3 | ＜5 | -0.56 | （-0.88，-0.24） | 0.0007 | 0 | 0.43 | 3.39 |
|  | 1 | ≥5 | -0.17 | （-0.39，0.06） | 0.15 | —— | —— | 1.45 |
| WHR | 4 | ＜5 | -0.96 | （-2.52，0.60） | 0.23 | 95 | 0.01 | 1.21 |
|  | 1 | ≥5 | 0.00 | （-0.23，0.23） | 1 | —— | —— | 0 |

**Supplementary Table 4.** Subgroup analysis of intervention frequencies.

| Outcomes | Amount | Subgroup  (Exercises) | SMD | 95%CI | P | Heterogeneity | | Z |
| --- | --- | --- | --- | --- | --- | --- | --- | --- |
|  |  |  |  |  |  | I^2^(%) | P |  |
| BMI | 4 | AE | -0.76 | （-1.74，0.21） | 0.13 | 89 | 0.01 | 1.53 |
|  | 4 | AE+RT | -0.17 | （-0.92，0.57） | 0.65 | 77 | 0.005 | 0.45 |
|  | 3 | Others | -0.16 | （-0.37，0.05） | 0.13 | 0 | 0.40 | 0.50 |
| BF% | 3 | AE | 0.42 | （-0.06，0.89） | 0.09 | 29 | 0.24 | 1.72 |
|  | 4 | AE+RT | -0.55 | （-1.58，0.47） | 0.29 | 87 | 0.01 | 1.06 |
|  | 4 | Others | -0.59 | （-1.32，0.13） | 0.11 | 92 | 0.01 | 1.60 |
| WC | 2 | AE | -1.71 | （-3.75，0.34） | 0.10 | 94 | 0.01 | 1.64 |
|  | 4 | AE+RT | -0.37 | （-0.67，-0.08） | 0.01 | 0 | 0.50 | 2.46 |
|  | 2 | Others | -0.23 | （-0.44，-0.02） | 0.03 | 0 | 0.37 | 2.12 |
| HC | 2 | AE | -0.68 | （-1.06，-0.30） | 0.0004 | 0 | 0.59 | 3.51 |
|  | 1 | AE+RT | -1.91 | （-2.36，-1.46） | 0.00001 | —— | —— | 8.28 |
|  | 1 | Others | -0.17 | （-0.39，0.06） | 0.15 | —— | —— | 1.45 |
| WHR | 2 | AE | -1.70 | （-6.13，2.73） | 0.45 | 98 | 0.01 | 0.75 |
|  | 2 | AE+RT | -0.19 | （-0.76，0.37） | 0.50 | 41 | 0.19 | 0.67 |
|  | 3 | Others | 0.18 | （-0.18，0.54） | 0.34 | 72 | 0.03 | 0.96 |

**Supplementary Table 5.** Subgroup analysis of intervention types.

| Outcomes | Study | SMD95% CI | P-Value | Tau2 | I^2^(%) |
| --- | --- | --- | --- | --- | --- |
| BMI | Chang-HoHa 2012 | -0.46[-0.87,-0.0] | 0.02 | 0.31 | 81 |
|  | Chih-Hui Chiu 2017 | -0.27[-0.59,0.04] | 0.09 | 0.15 | 67 |
|  | Eun-Dam Lee 2022 | \| -0.43[-0.84,-0.0] \| \| --- \| | 0.04 | 0.32 | 82 |
|  | Haitao Liu 2023 | \| -0.50[-0.91,-0.0] \| \| --- \| | 0.02 | 0.31 | 80 |
|  | Jing Sun 2020 | \| -0.46[-0.93,0.00] \| \| --- \| | 0.05 | 0.42 | 79 |
|  | Jun Zhao 2019 | \| -0.34[-0.73,0.05] \| \| --- \| | 0.08 | 0.28 | 79 |
|  | Meng Zhao 2024 | \| -0.36[-0.76,0.04] \| \| --- \| | 0.08 | 0.29 | 78 |
|  | Miao Wu 2022 | \| -0.43[-0.84,-0.0] \| \| --- \| | 0.04 | 0.32 | 82 |
|  | Mohamed Ahmed Said 2020 | \| -0.52[-0.91,-0.1] \| \| --- \| | 0.008 | 0.27 | 79 |
|  | Wenjiang Liu 2023 | \| -0.44[-0.89,0.01] \| \| --- \| | 0.06 | 0.39 | 82 |
|  | Xu Song 2024 | \| -0.41[-0.83,0.01] \| \| --- \| | 0.06 | 0.33 | 82 |
| BF% | Aoyao Zhang 2023 | \| -0.41 [-0.94, 0.13] \| \| --- \| | 0.14 | 0.61 | 88 |
|  | Chang-Ho Ha 2012 | \| -0.35 [-0.83, 0.14] \| \| --- \| | 0.16 | 0.50 | 89 |
|  | Chih-Hui Chiu 2017 | \| -0.43 [-0.92, 0.05] \| \| --- \| | 0.08 | 0.47 | 88 |
|  | Eun-Dam Lee 2022 | \| -0.32 [-0.81, 0.17] \| \| --- \| | 0.20 | 0.50 | 89 |
|  | Haitao Liu 2023 | \| -0.43 [-0.92, 0.06] \| \| --- \| | 0.09 | 0.49 | 88 |
|  | Jing Sun 2020 | \| -0.37 [-0.96, 0.22] \| \| --- \| | 0.22 | 0.77 | 89 |
|  | Jun Zhao 2019 | \| -0.17 [-0.60, 0.26] \| \| --- \| | 0.44 | 0.37 | 86 |
|  | Miao Wu 2022 | \| -0.34 [-0.82, 0.15] \| \| --- \| | 0.17 | 0.50 | 89 |
|  | Mohamed Ahmed Said 2020 | \| -0.44 [-0.91, 0.04] \| \| --- \| | 0.07 | 0.47 | 88 |
|  | Wenjiang Liu 2023 | \| -0.28 [-0.78, 0.21] \| \| --- \| | 0.27 | 0.50 | 87 |
|  | Xu Song 2024 | \| -0.13 [-0.53, 0.27] \| \| --- \| | 0.52 | 0.30 | 83 |
| WC | Chang-Ho Ha 2012 | \| -0.67 [-1.13, -0.21] \| \| --- \| | 0.004 | 0.29 | 83 |
|  | Chih-Hui Chiu 2017 | \| -0.35 [-0.53, -0.17] \| \| --- \| | 0.0002 | 0.01 | 9 |
|  | Jing Sun 2020 | \| -0.70 [-1.22, -0.17] \| \| --- \| | 0.010 | 0.38 | 79 |
|  | Jun Zhao 2019 | \| -0.61 [-1.09, -0.14] \| \| --- \| | 0.01 | 0.31 | 83 |
|  | Meng zhao 2024 | \| -0.60 [-1.10, -0.10] \| \| --- \| | 0.02 | 0.34 | 82 |
|  | Mohamed Ahmed Said 2020 | \| -0.69 [-1.15, -0.24] \| \| --- \| | 0.003 | 0.29 | 82 |
|  | Wenjiang Liu 2023 | \| -0.65 [-1.18, -0.11] \| \| --- \| | 0.02 | 0.39 | 83 |
|  | Xu Song 2024 | \| -0.63 [-1.11, -0.15] \| \| --- \| | 0.01 | 0.32 | 83 |
| HC | Chih-Hui Chiu 2017 | \| -0.77 [-1.58, 0.05] \| \| --- \| | 0.07 | 0.64 | 94 |
|  | Jing Sun 2020 | \| -0.88 [-1.64, -0.11] \| \| --- \| | 0.03 | 0.53 | 88 |
|  | Meng Zhao 2024 | \| -0.71 [-1.60, 0.17] \| \| --- \| | 0.11 | 0.75 | 94 |
|  | Wenjiang Liu 2023 | \| -0.38 [-0.68, -0.07] \| \| --- \| | 0.02 | 0.04 | 45 |
|  | Xu Song 2024 | \| -0.83 [-1.65, -0.02] \| \| --- \| | 0.05 | 0.64 | 94 |
| WHR | Aoyao Zhang 2023 | \| -0.53 [-1.21, 0.16] \| \| --- \| | 0.13 | 0.63 | 92 |
|  | Chih-Hui Chiu 2017 | \| 0.14 [-0.13, 0.41] \| \| --- \| | 0.30 | 0.06 | 64 |
|  | Jing Sun 2020 | \| -0.47 [-1.24, 0.30] \| \| --- \| | 0.23 | 0.81 | 93 |
|  | Meng Zhao 2024 | \| -0.51 [-1.15, 0.12] \| \| --- \| | 0.11 | 0.53 | 93 |
|  | Mohamed Ahmed Said 2020 | \| -0.30 [-0.90, 0.30] \| \| --- \| | 0.32 | 0.48 | 93 |
|  | Wenjiang Liu 2023 | \| -0.43 [-1.11, 0.24] \| \| --- \| | 0.21 | 0.61 | 93 |
|  | Xu Song 2024 | \| -0.40 [-1.03, 0.22] \| \| --- \| | 0.21 | 0.53 | 93 |

**Supplementary Table 6.** Sensitivity analysis results.
